# Impact of the WHO FCTC over the first decade: a global evidence review prepared for the Impact Assessment Expert Group

**DOI:** 10.1136/tobaccocontrol-2018-054389

**Published:** 2018-06-07

**Authors:** Janet Chung-Hall, Lorraine Craig, Shannon Gravely, Natalie Sansone, Geoffrey T Fong

**Affiliations:** 1 Department of Psychology, University of Waterloo, Waterloo, Ontario, Canada; 2 School of Public Health and Health Systems, Waterloo, Ontario, Canada; 3 Ontario Institute for Cancer Research, Toronto, Ontario, Canada

**Keywords:** global health, prevention, public policy

## Abstract

**Objective:**

To present findings of a narrative review on the implementation and effectiveness of 17 Articles of the WHO Framework Convention on Tobacco Control (FCTC) during the Treaty’s first decade.

**Data sources:**

Published reports on global FCTC implementation; searches of four databases through June 2016; hand-search of publications/online resources; tobacco control experts.

**Study selection:**

WHO Convention Secretariat global progress reports (2010, 2012, 2014); 2015 WHO report on the global tobacco epidemic; studies of social, behavioural, health, economic and/or environmental impacts of FCTC policies.

**Data extraction:**

Progress in the implementation of 17 FCTC Articles was categorised (higher/intermediate/lower) by consensus. 128 studies were independently selected by multiple authors in consultation with experts.

**Data synthesis:**

Implementation was highest for smoke-free laws, health warnings and education campaigns, youth access laws, and reporting/information exchange, and lowest for measures to counter industry interference, regulate tobacco product contents, promote alternative livelihoods and protect health/environment. Price/tax increases, comprehensive smoking and marketing bans, health warnings, and cessation treatment are associated with decreased tobacco consumption/health risks and increased quitting. Mass media campaigns and youth access laws prevent smoking initiation, decrease prevalence and promote cessation. There were few studies on the effectiveness of policies in several domains, including measures to prevent industry interference and regulate tobacco product contents.

**Conclusions:**

The FCTC has increased the implementation of measures across several policy domains, and these implementations have resulted in measurable impacts on tobacco consumption, prevalence and other outcomes. However, FCTC implementation must be accelerated, and Parties need to meet all their Treaty obligations and consider measures that exceed minimum requirements.

## Introduction

Tobacco use is a leading cause of premature mortality and disease burden worldwide, resulting in approximately seven million preventable deaths annually.^1–3^ It is estimated that if current trends continue, tobacco will kill more than eight million people globally each year by 2030, with 80% of premature deaths occurring in low-income and middle-income countries (LMICs).^4–7^

In response to the globalisation of the tobacco epidemic, the WHO Framework Convention on Tobacco Control (FCTC) was adopted by the World Health Assembly in 2003 and entered into force in 2005. The FCTC is one of the most widely adopted United Nations (UN) Treaties, with 181 Parties as of May 2018. It provides a comprehensive strategy for Parties to combat the tobacco epidemic and sets out a broad range of evidence-based measures to reduce tobacco demand (Articles 6–14) and supply (Articles 15–17).^8 9^

The year 2015 marked the tenth anniversary of the FCTC coming into force, as well as the introduction of the UN Sustainable Development Goals (SDGs), a comprehensive set of health-related goals and targets for all countries to achieve by 2030. Over the last decade, the prevalence of tobacco use has declined in countries with policies that align with or exceed the minimum requirements of the FCTC and its guidelines.^10–13^ Nevertheless, recent evidence suggests that many countries are not on track to achieve the WHO target of a 30% relative reduction in adult tobacco use by 2025.^14 15^

A decision by the FCTC Conference of the Parties (COP) at its sixth session in Moscow in October 2014 (FCTC/COP6(13)) established an independent group of seven experts to conduct an impact assessment to ‘examine the impact of the WHO FCTC on the implementation of tobacco control measures and on the effectiveness of its implementation’ over the first decade of the Convention.^16^ As of 2017, the WHO Convention Secretariat has published seven reports on global progress in FCTC implementation,^17–23^ and the WHO has published six reports that track the status of the global tobacco epidemic and policy interventions.^24^

Existing literature reviews of the FCTC’s impact focus on the evaluation of key measures to reduce the demand for tobacco: monitoring tobacco use; smoke-free laws; tobacco cessation interventions; health warnings; tobacco advertising, promotion and sponsorship (TAPS) bans; and tobacco tax increases. A systematic overview by Hoffman and Tan^25^ identified 59 systematic reviews summarising over 1150 primary studies (up to May 2015) on the impact of FCTC policies on tobacco use, second-hand smoke (SHS) exposure and primary health outcomes. Evidence was strongest for the effectiveness of smoke-free and tobacco taxation policies, followed by mass media campaigns and health warnings on the harms of tobacco use, and affordable smoking cessation treatment interventions; limited for advertising restrictions; and unavailable for monitoring tobacco use. A recent review of 41 studies (up to June 2017) on the effect of key demand-reduction measures on perinatal and child health found that smoke-free legislation was consistently associated with positive child health outcomes, including lower rates of preterm birth, and hospital admissions for childhood asthma and respiratory tract infections, with stronger associations for comprehensive bans than partial bans.^26^

It is estimated that nearly 22 million future premature smoking-attributable deaths were averted as a result of strong implementation of demand-reduction measures adopted by countries between 2007 and 2014.^27^ Consistent with this, Dubray *et al* 10 found that overall, countries with higher levels of implementation on these key measures experienced greater decreases in current tobacco smoking between 2006 and 2009. Similar findings on the positive effects of these demand-reduction measures on reducing smoking prevalence and cigarette consumption during 2007–2014 were found in another study by Ngo *et al*.^28^ A recent study by Gravely *et al* 29 found that increases in highest level implementations of the five key FCTC demand-reduction measures between 2007 and 2014 were significantly associated with a decrease in smoking prevalence between 2005 and 2015.

While there is a large evidence base for the effectiveness of these core demand-reduction measures, little is known about the impact of other FCTC policies, such as supply-reduction measures to reduce illicit tobacco trade, prohibit sales to and by minors and promote alternative livelihoods. Under decision FCTC/COP6(13),^30^ desk reviews of existing literature on the impact of the FCTC were mandated as a part of the work of the Expert Group (EG). In 2015–2016, the International Tobacco Control Policy Evaluation Project (ITC Project) conducted a global evidence review of the impact of the FCTC on the implementation of tobacco control legislation and the effectiveness of those implementations across a much broader set of FCTC policy domains. Further details on other literature reviews prepared for the EG as well as the methodology used by the EG to conduct the FCTC impact assessment are provided in this volume. In brief, the ITC global evidence review was prepared to inform the EG in deliberations at its first meeting (Geneva, August 2015). The global evidence review played a central role in the work of the EG by providing the context for the preparation of briefing materials on the status of FCTC implementation for the 12 country missions, and serving as a main evidence source for the EG’s report on their findings of the impact assessment to the COP at its seventh session (COP7; New Delhi, November 2016). The EG’s final report, ITC global evidence review and other relevant materials are available on the WHO Convention Secretariat website: http://www.who.int/fctc/cop/cop7/Documentation-Supplementary-information/en/.

This paper summarises the ITC global evidence review, which represents the most comprehensive overview and assessment of FCTC impact to date across the Treaty’s first decade. Given the limited time frame that was available (May–July 2015 for completion of literature review for EG’s first meeting on 10–11 August 2015; June 2016 for updates to correspond with the time of the EG’s submission of their final report of findings of the impact assessment to the COP on 29 June 2016), this review is not a systematic review of all available evidence on the implementation and effectiveness of policies called for under the 17 FCTC Articles. Rather, it is a narrative review that aims to provide a qualitative synthesis of evidence on whether the FCTC has increased and strengthened the implementation of tobacco control policies under the 17 Articles of the Convention and the effectiveness of those measures. A narrative review provides a qualitative summary of primary studies on a research question, covers a wide range of issues on a topic, and allows for the inclusion of a broad range of evidence sources that use different study designs and report diverse outcomes,^31 32^ and is thus well suited for the purpose of the current review.

## Methods

This narrative review was conducted across 17 substantive Articles of the FCTC, where impact assessment was appropriate (table 1 provides a brief description of each Article, and further details are provided in online supplementary table S1).^i^ The 2010, 2012 and 2014 global progress reports on FCTC implementation prepared by the WHO Convention Secretariat^20–22^ and the 2015 WHO report on the global tobacco epidemic^33^ served as the primary sources to assess global progress and key challenges in FCTC implementation. Relevant data from published studies and policy reports were also included. Based on an overall assessment of available sources (up to June 2016) on changes in the total number of countries/FCTC Parties who reported the implementation of Treaty provisions over time, the level of progress for each Article was categorised as higher (significant and rapid progress), intermediate (some progress but slower overall rate, with advancements often limited to partial implementation) or lower (some momentum to support the development of measures but slow progress).

10.1136/tobaccocontrol-2018-054389.supp1 Supplementary file 1

**Table 1:** Brief description of the Framework Convention on Tobacco Control (FCTC) Articles included in this review

FCTC Article	Description
Article 5.3	Protect tobacco control policies against industry interference.
Article 6	Price and tax measures to reduce tobacco consumption, including raising the price of tobacco products through taxation, prohibiting/restricting tobacco sales to international travellers and dedicating tobacco tax revenues to fund tobacco control.
Article 8	Protection from exposure to tobacco smoke in indoor public places, workplaces, public transport and other public places.
Article 9	Regulation of tobacco product contents through testing and measuring contents and emissions of tobacco products.
Article 10	Regulation of tobacco product disclosures by requiring tobacco manufacturers and importers to disclose information about contents, toxic constituents and emissions of their products.
Article 11	Require health warnings on tobacco product packaging and prohibit misleading tobacco packaging and labelling.
Article 12	Use all available communication tools to promote education, communication, training and public awareness of tobacco control issues.
Article 13	Enforce comprehensive bans on all forms of tobacco advertising, promotion and sponsorship.
Article 14	Promote tobacco cessation and provide treatment for dependence through healthcare providers, and accessible, low-cost interventions.
Article 15	Eliminate all forms of illicit trade in tobacco products, including smuggling, illicit manufacturing and counterfeiting.
Article 16	Prohibit sales of tobacco products to and by minors, including a ban on the sale of tobacco products at point of sale, restrictions on accessibility to tobacco vending machines and ban on the sale of single cigarettes or small packs.
Article 17	Promote economically viable alternatives for tobacco workers, growers and individual sellers.
Article 18	Protect the environment and health of persons with respect to the cultivation and manufacturing of tobacco.
Article 19	Legislative action to deal with criminal and civil liability, including compensation where appropriate.
Article 20	Research, surveillance and exchange of information on tobacco control, including patterns of, determinants and outcomes of tobacco consumption.
Article 21	Require Parties to submit periodic reports on implementation of the Convention.
Article 22	International cooperation to promote the transfer of technical, scientific and legal expertise and technology to establish and strengthen national tobacco control strategies.

Published studies and grey literature on the effectiveness of FCTC measures were identified from searches of four electronic databases: PubMed, Cochrane Library, Scopus and Google Scholar. Independent searches were conducted by three authors (JC-H, SG, NS) and screened by all coauthors between May and July 2015. Searches were updated by the lead author (JC-H) in June 2016 and reviewed by the second author (LC). Tobacco control experts (online supplementary table S2) were also contacted to identify further sources. Studies were eligible for inclusion if they were primary or secondary research on the social, behavioural, health economic and/or environmental impacts of FCTC tobacco control policies. Searches were conducted for the full time period available up to June 2016. Non-English-language studies and tobacco industry-funded research were excluded. A broad range of general and FCTC Article-specific search terms were used for all databases to capture as many publications on the effectiveness of FCTC measures as possible. Additional materials were identified by hand-searching selected peer-reviewed publications and grey literature, and scanning online resources created by leading tobacco control advocacy/research groups, such as the Campaign for Tobacco-Free Kids, Framework Convention Alliance, University of California San Francisco Truth Tobacco Industry Documents and Tobacco Tactics.

10.1136/tobaccocontrol-2018-054389.supp2 Supplementary file 2

A total of 128 studies, identified in consultation with a panel of tobacco control experts, were included in this review.

## Results

### Impact of FCTC on tobacco control legislation

**Figure 1:**
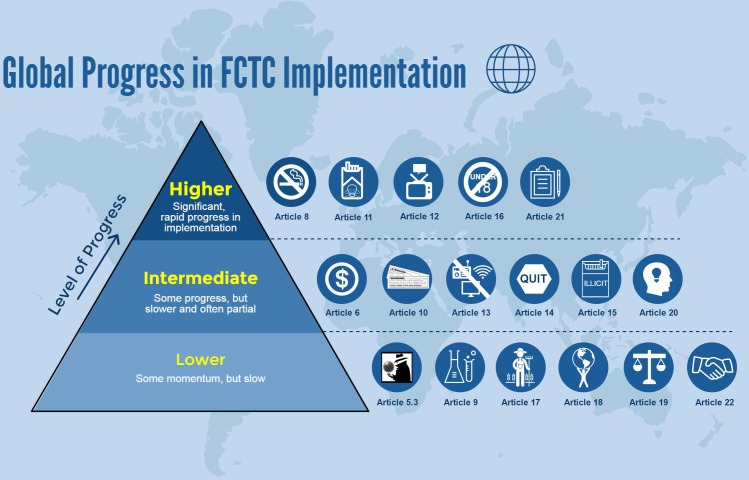
Global progress in Framework Convention on Tobacco Control (FCTC) implementation based on available sources up to June 2016.

FCTC implementation reports submitted by Parties to the Convention Secretariat show progress in the implementation of tobacco control legislation since the Convention came into force in 2005. However, there is considerable variability in the overall rate and extent of progress in the implementation of tobacco control legislation across countries and policy domains, with limited progress in the implementation of strong policies in many LMICs. Figure 1 summarises overall progress in the implementation of measures for each of the 17 FCTC Articles.

The FCTC has contributed to significant and rapid progress in the implementation of the following:

1. Comprehensive smoke-free laws for indoor workplaces, restaurants and bars (Article 8).^22 33 34^
2. Larger, pictorial health warnings on cigarette packages (Article 11).^35–38^
3. Mass media education campaigns on the health risks of tobacco consumption and exposure to tobacco smoke, and the benefits of quitting (Article 12).^20 22^
4. Bans on the sale of tobacco products to and by minors (Article 16).^22 39 40^

Since the first reporting cycle in 2007, there has also been a steady increase in the number of Parties that have submitted FCTC implementation reports in accordance with Article 21.^22^

The FCTC has contributed to some progress in the implementation of the following:

1. Tobacco price and tax increases,^22 33^ simplified tax systems^41–43^ and tax measures that account for inflation (Article 6).^44–47^
2. Disclosure of information on the contents and emissions of tobacco products (Article 10).^22^
3. Comprehensive TAPS bans (Article 13).^33^
4. Cessation services (Article 14).^20 22 33^
5. Measures to counter illicit tobacco trade (Article 15).^22^
6. Programmes for tobacco-related research, surveillance and information exchange (Article 20).^22 48^

However, overall global progress in these policy domains has been slow and advancements have often been limited to partial implementation.

Finally, the FCTC has generated momentum, in a small number of countries, to support the development of measures to:

1. Protect tobacco control policies from industry interference (Article 5.3).^22^
2. Regulate the contents and emissions of tobacco products (Article 9).^22^
3. Promote economically viable alternatives for tobacco farmers (Article 17).^20 22 49 50^
4. Address the health and environmental impacts related to the cultivation and manufacture of tobacco (Article 18).^22 51^
5. Allow for legislative action against the tobacco industry (Article 19).^22 52^
6. Facilitate international cooperation (Article 22).^21 22^

### Ongoing challenges to the implementation of the FCTC

The FCTC has generally had a positive impact on tobacco control. Nevertheless, there are a number of ongoing challenges to the effective implementation of the Convention.

#### Tobacco industry interference

The tobacco industry has a long-standing history of using direct and indirect tactics to obstruct, delay or weaken the implementation of FCTC policies, including smoke-free laws, tobacco marketing bans, and price and tax measures.^53–57^ Although an increasing number of countries have taken steps to prevent tobacco industry interference (TII) with policymaking in recent years, no country has fully implemented measures to protect public health policy from TII at the best practice level as recommended under Article 5.3 to date.^3 23 33 58^ As a result, TII continues to be the largest barrier to FCTC implementation worldwide. The tobacco industry continues to use strategies that are in violation of Article 5.3 guidelines, including government partnerships,^59 60^ front groups^61–63^ and corporate social responsibility activities,^64^ as well as strategies that are not directly covered by Article 5.3 guidelines to interfere with policymaking. For example, the industry has used trade and investment agreements to challenge legislation for plain packaging in Australia and larger health warnings in Uruguay.^57 65–68^ The industry has also established ‘Better Regulation Agendas’ to promote their interests and block the implementation of evidence-based policies, such as the 2014 Tobacco Product Directive in the European Union and plain packaging in the UK.^63 69 70^

It is encouraging, however, that such industry tactics have been unsuccessful. Notably, legal challenges to legislation for plain packaging in Australia and the UK, and 80% front and back pictorial warnings and single brand presentation in Uruguay, have all been dismissed by domestic and international courts/tribunals.^71–76^ These landmark rulings reinforce that governments have the right to implement FCTC measures for the protection of public health and are expected to set a strong precedent for the introduction of similar legislation in other countries.^77 78^

#### Lack of guidelines and ineffective implementation of existing guidelines

In general, progress in FCTC implementation has been more rapid and comprehensive for Articles with existing guidelines and specified timelines for implementation of certain provisions. Formal guidelines have not yet been adopted to assist Parties to meet their Treaty obligations under Articles 9, 10, 15 and 17–22. Selective and incomplete implementation of existing guidelines allows the tobacco industry to take advantage of loopholes in existing legislation, thus weakening the policy impact. For example, few countries earmark tobacco tax revenues for health purposes; prohibit smoking in private workplaces, pubs and bars, and private motor vehicles; have health warnings covering more than 50% of the package; prohibit tobacco displays at point of sale; and mandate the recording of patients’ tobacco use in medical notes.^22 33 79^

#### Insufficient capacity and lack of financial support

In many countries, there is limited capacity for tobacco control at the national level. For example, Parties have identified the lack of capacity for testing contents of tobacco products and national data collection as barriers to the implementation of Articles 9 and 21, respectively. As of 2014, only 5 of 130 reporting Parties have established training programmes and strategies that aim to strengthen tobacco control capacity, as called for under Article 20.^22^ In most countries, governments provide limited (if any) financial support for core FCTC measures, including cessation services and tobacco dependence treatment,^33 80^ alternative livelihood programmes,^81–84^ measures for the protection of the environment and health of tobacco workers,^22 51^ and tobacco-related research programmes.^48^

#### Poor enforcement

In the vast majority of countries, there are weak enforcement mechanisms to ensure compliance with tobacco control policies. For example, many Parties continue to experience enforcement-related difficulties for smoke-free laws, TAPS bans and youth access laws.^21 22 37^

### Effectiveness of FCTC-compliant tobacco control measures

There is a growing body of research on the effectiveness of tobacco control measures that align with the FCTC and its existing guidelines. This narrative review included 128 studies on the effectiveness of FCTC measures up to June 2016 (online supplementary table S3). Overall, studies on the impact of price and tax increases (n=6),^22 44 85–88^ comprehensive smoke-free policies (n=17),^13 89–104^ health warnings (n=25),^105–129^ comprehensive TAPS bans (n=14)^130–143^ and cessation interventions (n=19)^79 144–161^ consistently found that these are among the most effective strategies to reduce tobacco consumption/prevalence and tobacco-related health risks, and encourage quitting. Studies conducted in high-income countries (HICs) also provide strong evidence that mass media campaigns (n=14)^162–175^ and well-enforced measures to restrict youth access to tobacco products (n=10)^176–185^ are effective for preventing smoking initiation, decreasing smoking prevalence and promoting cessation.

10.1136/tobaccocontrol-2018-054389.supp3 Supplementary file 3

A small number of case studies provide evidence for the effectiveness of coordinated national strategies to combat illicit trade in the UK, Spain and Kenya (n=4)^33 186–188^; profitability of small-scale alternative crop programmes in China, Kenya, Zimbabwe, Malawi, Bangladesh and Brazil (n=9)^189–197^; and reductions in green tobacco sickness among tobacco workers who used personal protective equipment in the USA, India and Malaysia, as well as those who were exposed to a public education campaign on the risks of tobacco harvesting in the USA (n=7).^198–204^ One study found that regular monitoring of tobacco use is associated with a decrease in smoking rates over time.^10^ Two reviews summarised the use of the FCTC and its guidelines in legislation and litigation, and showed an increase in the number of countries who have used the Treaty to support new tobacco control policies and to defend legislation against industry challenges.^52 205^

No studies evaluating the impact of measures for prevention of industry interference, regulation of contents of tobacco products, and facilitation of information exchange and cooperation were identified.

## Discussion

This narrative review is the first to synthesise global research evidence on the impact of the FCTC after its first 10 years. It is the broadest assessment to date of whether the implementation of tobacco control legislation across 17 substantive Articles could be attributed to the FCTC, and whether implementation of those policies was linked to subsequent changes in tobacco consumption, prevalence and other tobacco-related outcomes.

The findings of the review were integral to the work of the EG, as they provided background and context for the country missions, and evidence that informed the judgements of the EG in their report to COP7 on the outcome of the impact assessment and recommendations on how to strengthen FCTC impact. Leading tobacco control experts were engaged in the literature review process and made contributions to gathering and reviewing evidence on the effectiveness of the implementation of the Convention.

The FCTC impact assessment contributed to building the foundation for shifting the focus of the COP from increasing ratification and guideline development towards actions to encourage accelerated implementation of the FCTC, including the establishment of an implementation working group under a decision adopted at COP7 (FCTC/COP7(13)).^206^

Overall, this review shows that tobacco control policies are effective when they are implemented according to the Treaty and its guidelines. However, the overall rate and extent of global progress in the implementation of the provisions of the Convention remain uneven across countries and policy domains. Among 17 FCTC Articles, the greatest progress has been achieved in the implementation of smoke-free laws, health warnings on tobacco packaging, antitobacco mass media campaigns, youth access laws and reporting/exchange of information. On the other hand, progress in all other policy domains has been slow, particularly for the implementation of measures to counter industry interference, regulate tobacco product contents, promote alternative livelihoods and protect the environment and health of tobacco workers.

Only a small number of reporting Parties have taken liability action against the tobacco industry over the last decade. It is encouraging to note that the FCTC Treaty text and guidelines have been explicitly cited by a growing number of countries to support new tobacco control measures. The FCTC has also been successfully used as a legal instrument to defend Parties against industry challenges to tobacco control measures, including plain packaging in Australia, and 80% pictorial warnings and single brand presentation in Uruguay.

Although the FCTC has played an important role in driving global progress in the implementation of tobacco control policies over the last decade, there are ongoing challenges to the effective implementation of the Treaty. First, in order to achieve the WHO global target of a 30% reduction in tobacco use by the year 2025, progress in many policy domains needs to be accelerated. Second, the tobacco industry continues to be the greatest threat to the implementation of the FCTC. In 2018, the WHO Convention Secretariat and the Global Center for Good Governance in Tobacco Control (a WHO FCTC Knowledge Hub on Article 5.3) published reports that identified best practices for effective implementation of FCTC Article 5.3 and its guidelines at the country and global level.^207 208^ There is an urgent need for Parties to implement these measures to eliminate industry interference with policymaking. Finally, long-term sustainable solutions to strengthen capacity, financial support and resources, and enforcement are required to assist Parties to meet their Treaty obligations.

A growing body of research indicates that tobacco control measures that align with the FCTC and its guidelines are effective. This review found strong international evidence that price and tax increases, comprehensive smoke-free policies, pictorial health warnings, comprehensive TAPS bans and cessation interventions are among the most effective strategies to reduce tobacco consumption and tobacco-related health risks, and encourage quitting. We also found that mass media campaigns and well-enforced measures to restrict youth access to tobacco products are consistently associated with decreased smoking initiation and smoking prevalence, and increased cessation in HICs. These results are consistent with several recent studies based on global data^28 209–211^ and systematic reviews^212–216^ on the effect of FCTC policies on tobacco-related outcomes, including improved health, decreased smoking prevalence and consumption, decreased SHS exposure, and increased smoking cessation.

On the other hand, there are still significant research gaps on the impact of FCTC measures in several key policy domains. In the vast majority of countries, the development and implementation of measures to prevent industry interference, regulate tobacco product contents and disclosures, promote economically viable alternatives, protect the environment and health, encourage liability action against the industry, and promote cooperation are still in the early stages. It will be important for future research to evaluate the effectiveness of measures in these areas as they are adopted. Finally, there is a paucity of research that has examined the impact of the FCTC by gender and among disadvantaged groups.

This global review has several limitations. First, our summary of global progress in FCTC implementation is largely based on Parties’ self-reports that are not systematically evaluated for consistency with implemented laws, regulations or national strategies/action plans. Furthermore, FCTC implementation reports do not require Parties to submit information on their use of implementation guidelines.^23^ Second, we did not analyse whether progress in policy implementation and subsequent public health impact is directly due to the FCTC or other factors. It is likely that changes are generated by the FCTC in combination with other country-specific factors, such as political climate, strength of tobacco control advocacy community, policy compliance and enforcement, and pre-existing legislation prior to FCTC ratification. However, the relative impact of the FCTC will vary by country. For example, a recent analysis of daily smoking prevalence estimates in 195 countries from 1990 to 2015 found that a greater percentage achieved significant annualised rates of decline in smoking prevalence from 1990 to 2005 (before FCTC) than from 2005 and 2015 (after FCTC).^2^ Moreover, there are non-Parties to the FCTC that have implemented strong national tobacco control policies, such as comprehensive smoke-free laws, large graphic health warnings and high tobacco taxes in Argentina, and antitobacco mass media campaigns and accessible tobacco dependence treatment interventions in the USA.^3^ Third, given the time constraints for completion of the literature review, we did not conduct a systematic review of all empirical evidence on the effectiveness of FCTC measures. Our literature searches were restricted to four databases, published data and English-language sources. We consulted with seven tobacco control experts to identify any key sources missed by our searches; however, their expertise did not cover all 17 FCTC Articles included in this review. Future systematic reviews are needed to synthesise all available evidence on the impact of FCTC policies on tobacco prevalence and consumption, and other outcomes. Fourth, we did not assign quality ratings to sources on FCTC policy impact to prioritise the selection of sources for the current review. Finally, we did not use standardised criteria to categorise the level of progress in FCTC implementation for the 17 Articles included in this review. However, the overall pattern of our findings on global progress in the implementation of FCTC policies is comparable with the latest results of the WHO Convention Secretariat’s 2016 global progress report on FCTC implementation across 16 Articles,^23^ as well as the WHO 2017 report on the global implementation of core demand-reduction measures.^3^

## Conclusion

This narrative review summarises evidence on FCTC impact over its first 10 years. The FCTC has served as a powerful tool to initiate, support and advance national, regional and global tobacco control efforts. The effectiveness of core demand-reduction policies is well established, and emerging evidence suggests that strong implementation of these measures can lead to significant reductions in tobacco use.^10 28 29^ It is now time for Parties to build on achievements and to address gaps in policy implementation and research, especially in LMICs. The 2030 Agenda for Sustainable Development recognises tobacco control as a critical component to achieve all 17 SDGs. In order to change the current trajectory of the global tobacco epidemic and meet the SDG targets, Parties need to accelerate the implementation of all FCTC provisions, in combination with systematic evaluation of policy effectiveness.

**What this paper adds**

- This narrative review synthesised evidence on the impact of the Framework Convention on Tobacco Control (FCTC) across 17 substantive Articles over its first 10 years.
- This narrative evidence review found that there has been an increase in the implementation of tobacco control legislation since the FCTC came into force, but progress varies across countries and policy domains.
- The FCTC is a powerful legal instrument that can be used by countries to support new tobacco control measures and to defend against industry challenges to legislation.
- There is strong international evidence that FCTC-compliant measures are effective.
- Significant gaps exist for both the implementation and evaluation of measures to counter industry interference (Article 5.3), regulate tobacco product contents (Article 9), promote alternative livelihoods (Article 17), protect the environment and health of tobacco workers (Article 18), and promote cooperation (Article 22).
- To achieve the Sustainable Development Goals to strengthen FCTC implementation and reduce premature mortality from non-communicable diseases, Parties need to fulfill their Treaty obligations, implement measures that go beyond the minimum provisions of the Convention and eliminate industry interference.

## Footnotes

i This narrative review did not include FCTC Articles for which impact assessment was not relevant: introduction (Articles 1–3); objective, guiding principles and general obligations (Articles 3–5); institutional arrangements and financial resources (Articles 23–26); settlement of disputes (Article 27); development of the Convention (Articles 28–29); and final provisions (Articles 30–38).
