# Supplementary material for: Impact of the WHO FCTC over the first decade: a global evidence review prepared for the Impact Assessment Expert Group
Source: Tob Control. 2018 Jun 7;28(Suppl 2):s119–28. doi: 10.1136/tobaccocontrol-2018-054389 (PMC6589489; doi:10.1136/tobaccocontrol-2018-054389)
Supplement: Supplementary file 1 [file tobaccocontrol-2018-054389supp001.pdf]

Table S1. Summary of FCTC articles and key provisions of their guidelines for implementation

FCTC Article	Implementation guidelines	Description of FCTC article and key provisions of existing guidelines
Article 5.3 – Protection of public health policies with respect to tobacco control from commercial and other vested interests of the tobacco industry	Guidelines adopted November 2008	Protect public health policies with respect to tobacco control from commercial and other vested interests of the tobacco industry in accordance with national law.<br><br>Guidelines recommend that Parties educate government and public about the dangers of tobacco products and industry interference with tobacco control policies; limit interactions with industry and ensure transparency of any interactions; reject government partnerships with the tobacco industry; prohibit industry involvement in policymaking; prohibit industry involvement in youth, public education, or other tobacco control initiatives; require provision of transparent and accurate information from industry; denormalize and regulate "socially responsible" activities by the industry; and ensure equal treatment of State-owned and any other tobacco industry.<br><br>http://www.who.int/fctc/guidelines/adopted/article_5_3/en/
Article 6 – Price and tax measures to reduce the demand for tobacco	Guidelines adopted October 2014	Adopt pricing and taxation measures in order to reduce tobacco consumption.<br><br>Guidelines recommend that Parties implement specific or mixed excise with minimum specific tax floor over purely ad valorem systems; establish coherent long-term tax policies to achieve health and fiscal objectives; tax all tobacco products equally and implement systems to minimize users' downward shifting to less expensive products; consider effective anti-forestalling measures to prevent/reduce tax avoidance; require fiscal markings to increase compliance with tax laws; dedicate tax revenue to tobacco control activities; adjust taxes for inflation and income growth on a regular basis to reduce product affordability; and design efficient and effective tax collection system.<br><br>http://www.who.int/fctc/guidelines/adopted/Guidelines_article_6.pdf?ua=1
Article 8 – Protection from exposure to tobacco smoke	Guidelines adopted July 2007	Adopt national legislation for protection from exposure to tobacco smoke in indoor public places, workplaces, public transport, and, as appropriate, other public places.<br><br>Guidelines recommend that Parties adopt measures to provide universal protection from tobacco smoke with no exemptions for ventilation and air filtration technology, and use of

FCTC Article	Implementation guidelines	Description of FCTC article and key provisions of existing guidelines
		designated smoking areas; extend comprehensive smoking bans to outdoor and quasi-outdoor spaces.<br><br>http://www.who.int/fctc/guidelines/adopted/article_8/en/
Article 9 – Regulation of the contents of tobacco products	Partial guidelines adopted November 2010, with amendments November 2012	Implement measures for the testing, measuring, and regulation of the contents and emissions of tobacco products.<br><br>Partial guidelines recommend that Parties prohibit/restrict ingredients that may be used to increase the palatability of tobacco products, those with coloring properties that increase product appeal, or that may create the impression of health benefits; require industry to provide proof of accreditation for testing laboratories; and require that cigarettes comply with a reduced ignition propensity standard.<br><br>http://www.who.int/fctc/guidelines/adopted/article_9and10/en/
Article 10 – Regulation of tobacco product disclosures	Partial guidelines adopted November 2010, with amendments November 2012	Implement measures that require manufacturers and importers of tobacco products to disclose information about the contents and emissions of tobacco products to governmental authorities and the public.<br><br>Guidelines recommend that Parties require tobacco manufacturers and importers to disclose to governmental authorities information about product ingredients, design features, company information, and sales volume; and to make information about toxic constituents and emissions disclosed to government available to the public.<br><br>http://www.who.int/fctc/guidelines/adopted/article_9and10/en/
Article 11 – Packaging and labelling of tobacco products	Guidelines adopted November 2008	Adopt, within three years after entry into force of the FCTC for a Party, measures to prohibit misleading tobacco packaging and labelling; ensure that tobacco product packages display health warnings and messages (that may include pictures) that cover at least 50% and no less than 30% of the principal display areas; and ensure that tobacco packaging contains information on product constituents and emissions.<br><br>Guidelines recommend that Parties prohibit packaging and labelling that promotes a tobacco product by means that are false, misleading, deceptive, or likely to create an

FCTC Article	Implementation guidelines	Description of FCTC article and key provisions of existing guidelines
		erroneous impression about product characteristics, health effects, hazards or emissions, including the use of any term, descriptor, trademark or figurative, or other sign that creates the false impression that a product is less harmful than others (e.g., terms such as “low tar”, “light”, “ultra-light” or “mild”); implement rotating pictorial and text health warnings that cover more than 50% of the principal display areas; prohibit the display of figures for emission yields; and adopt plain or standardized packaging.<br><br>http://www.who.int/fctc/guidelines/adopted/article_11/en/
Article 12 – Education, communication, training, and public awareness	Guidelines adopted November 2010	Promote and strengthen public awareness of tobacco control issues, using all available communication tools, as appropriate.<br><br>Guidelines recommend that Parties establish an infrastructure and build capacity to raise public awareness of tobacco control issues and promote social change using all available means, including international collaboration and involvement of members of civil society that do not have ties with the tobacco industry; ensure that the public has free and universal access to a wide range of information on tobacco industry strategies/activities and its products; and monitor and evaluate implemented measures.<br><br>http://www.who.int/fctc/guidelines/adopted/article_12/en/
Article 13 – Tobacco advertising, promotion, and sponsorship (TAPS)	Guidelines adopted November 2008	Implement, within five years after entry into force of the FCTC for a Party, a comprehensive ban on all forms of TAPS.<br><br>Guidelines recommend Parties ensure that TAPS bans do not allow for any exemptions; prohibit tobacco vending machines; adopt plain packaging; prohibit Internet sales of tobacco; prohibit “brand stretching” and “brand sharing”; ban corporate “socially responsible” activities; prevent use of journalistic, artistic or academic expression or social/political commentary for the tobacco promotion; prohibit use of identifiable tobacco brands or imagery in entertainment media; prohibit cross-border TAPS; and promote public awareness of need to eliminate TAPS.<br><br>http://www.who.int/fctc/guidelines/adopted/article_13/en/

FCTC Article	Implementation guidelines	Description of FCTC article and key provisions of existing guidelines
Article 14 – Demand reduction measures concerning tobacco dependence and cessation	Guidelines adopted November 2010	Promote cessation of tobacco use and treatment for tobacco dependence.<br><br>Guidelines recommend that Parties provide cessation support and treatment in all health care by trained providers; require recording of patients’ tobacco use in medical notes; establish infrastructure and create an environment that prompts quit attempts by establishing a national cessation strategy and treatment guidelines, addressing the issue in health care workers, integrating brief advice into existing health care systems, and creating capacity for widely accessible, evidence-based, and affordable tobacco cessation interventions; monitor and evaluate strategies and programmes; and promote international cooperation.<br><br>http://www.who.int/fctc/guidelines/adopted/article_14/en/
Article 15 – Illicit trade in tobacco products	Guidelines have not yet been developed	Eliminate all forms of illicit trade in tobacco products, including smuggling, illicit manufacturing, and counterfeiting.<br><br>Protocol to Eliminate Illicit Trade in Tobacco Products was adopted November 2012, and will enter into force on the 90th day following ratification, acceptance, approval or accession by 40 FCTC Parties. As of May 2018, there are 54 Signatories and 36 Parties to the Protocol.
Article 16 – Sales to and by minors	Guidelines have not yet been developed	Prohibit sale of tobacco products to minors (persons under the age set by domestic law, national law, or 18).
Article 17 – Provision of support for economically viable alternatives	Draft guidelines adopted October 2014	Promote, as appropriate, economically viable alternatives for tobacco workers, growers, and individual sellers.<br><br>http://www.who.int/fctc/guidelines/adopted/Policy_options_reccommendations_Articles17_18_COP6.pdf?ua=1
Article 18 – Protection of the environment and the health of persons	Draft guidelines adopted October 2014	Protect the environment and health of persons in relation to the environment in respect of tobacco cultivation and manufacture within their respective territories.<br><br>http://www.who.int/fctc/guidelines/adopted/Policy_options_reccommendations_Articles17_18_COP6.pdf?ua=1

FCTC Article	Implementation guidelines	Description of FCTC article and key provisions of existing guidelines
Article 19 – Liability	Guidelines have not yet been developed	Take legislative action to deal with criminal and civil liability, including compensation where appropriate; and promote international cooperation to assist in legal proceedings and information exchange.
Article 20 – Research, surveillance, and exchange of information	Guidelines have not yet been developed	Establish national, regional and global health surveillance programmes; implement and promote tobacco control-related research; facilitate cooperative exchange of tobacco control-related information; and provide support to developing country Parties and Parties with economies in transition to meet their obligations under Article 20.
Article 21 – Reporting and exchange of information	Guidelines have not yet been developed	Submit to the COP, through the Secretariat, periodic reports on implementation of the Convention. Each Party is obligated to make its initial report within two years of the entry into force of the FCTC for that Party.
Article 22 – Cooperation in the scientific, technical, and legal fields and provision of related expertise	Guidelines have not yet been developed	Cooperate directly or through competent international bodies to strengthen capacity for transfer of technical, scientific, and legal expertise and technology to establish and strengthen national tobacco control strategies, plans, and programmes.
