# Supplementary material for: Impact of the WHO FCTC over the first decade: a global evidence review prepared for the Impact Assessment Expert Group
Source: Tob Control. 2018 Jun 7;28(Suppl 2):s119–28. doi: 10.1136/tobaccocontrol-2018-054389 (PMC6589489; doi:10.1136/tobaccocontrol-2018-054389)
Supplement: Supplementary file 2 [file tobaccocontrol-2018-054389supp002.pdf]

Table S2. Panel of consulted tobacco control experts

**Tobacco Control Expert**	**Affiliation**
Dr. Gary J. Fooks	Aston University
Dr. Anna B. Gilmore	University of Bath
Dr. Richard O'Connor	Roswell Park Cancer Institute
Rob Cunningham	Canadian Cancer Society
Dr. Martin Raw	International Centre for Tobacco Cessation, University of Nottingham
Dr. Jeffrey Drope	American Cancer Society
Dr. Natacha Lecours	International Development Research Centre
