# Supplementary material for: Impact of the WHO FCTC over the first decade: a global evidence review prepared for the Impact Assessment Expert Group
Source: Tob Control. 2018 Jun 7;28(Suppl 2):s119–28. doi: 10.1136/tobaccocontrol-2018-054389 (PMC6589489; doi:10.1136/tobaccocontrol-2018-054389)
Supplement: Supplementary file 3 [file tobaccocontrol-2018-054389supp003.pdf]

Table S3. Summary of key findings from 128 studies on the impact of FCTC policies.

FCTC Article	Policy evaluation	Source	Key findings on policy impact	Overall evidence for policy effectiveness
**Article 5.3 – Protection of public health policies with respect to tobacco control from commercial and other vested interests of the tobacco industry**	n/a	n/a	n/a	None to date
**Article 6 – Price and tax measures to reduce the demand for tobacco**	Cigarette tax increase	Warren et al.[85]<br>WHO Convention Secretariat [22]	Decrease in cigarette sales	Strong
		Yürekli et al.[86]<br>Waters et al. [87]	Increase in government tax revenues	
		Van Walbeek [88]<br>Dorotheo et al. [44]<br>WHO Convention Secretariat [22]	Decrease in smoking prevalence	
**Article 8 – Protection from exposure to tobacco smoke**	Comprehensive smoke-free law	WHO WPR & ITC Project [89]<br>Fong et al. [90] [91]<br>Cooper et al. [92]	Reduction in smoking in indoor public places	Strong
		Brennan et al. [93]<br>Avila-Tang et al. [94]<br>Connolly et al. [95]<br>Hyland et al. [96]	Reduction in levels of air pollutants and improvement in air quality	
		Goodman et al. [97]<br>Frazer et al. [13]<br>Stallings-Smith et al. [98]<br>Sebrié et al. [99]<br>Sebrié et al. [100]<br>Pell et al. [101]<br>Tan & Glantz [102]<br>Been et al. [103]	Improvement in health outcomes	
		Fowkes et al. [104]<br>Fong et al. [90]	Increase in quitting among smokers	
**Article 9 – Regulation of the contents of tobacco products**	n/a	n/a	n/a	None to date
**Article 10 – Regulation of tobacco product disclosures**	n/a	n/a	n/a	None to date

FCTC Article	Policy evaluation	Source	Key findings on policy impact	Overall evidence for policy effectiveness
**Article 11 – Packaging and labelling of tobacco products**	Large pictorial health warnings (>50%)	Gravely et al. [105]	Increase in adult smokers' noticing and reading warnings; thinking about risks of smoking; thinking about quitting; avoiding warnings; and forgoing a cigarette	Strong
	Pictorial (graphic) health warnings	Hammond [106]	Increase in adult smokers' noticing of warnings and cessation behaviour; and decrease in uptake of smoking among youth	
		Hammond et al. [107]<br>Hammond et al. [108]	Promotes cessation among adult smokers	
		Hammond et al. [109]	Pictorial warnings perceived by adult and youth smokers as more realistic and effective vs. illustrations/cartoons	
		Huang et al. [110]	Decrease in adult smoking prevalence	
		Hammond et al. [111]	Increase in adult smokers' knowledge of harms of smoking	
		Borland et al. [112]	Increase in adult smokers' noticing of warnings, thinking about health risks and quitting, forgoing cigarettes, and avoiding warnings	
		ITC Project [113]<br>Fathelrahman et al. [114]	Increase in adult smokers' thinking about health risks of smoking and quitting behaviour	

FCTC Article	Policy evaluation	Source	Key findings on policy impact	Overall evidence for policy effectiveness
		Chiosi et al. [115]	Greater knowledge of harms of smoking among adult smokers	
	Inclusion of quitline number on cigarette packaging	Miller et al. [116]<br>Grigg [117]<br>Wilson et al. [118]	Increase in use of national quitlines	
	Plain packaging for cigarettes	British Heart Foundation & ITC Project [119]<br>Hammond [120]<br>Chantler [121]<br>Moodie et al. [122]<br>Wakefield et al. [123]<br>Yong et al. [124]<br>Dunlop et al. [125]<br>Durkin et al. [126]<br>Young et al. [127]<br><br>Australian Department of Health [128]<br>Kmietowicz [129]	Reduces false health beliefs; increases noticeability of health warnings; enhances cognitive processing of health warnings; greater avoidance of warnings; reduces appeal of packaging; reduces consumer demand; and/or promotes smoking cessation<br><br>Decrease in adult smoking prevalence	
**Article 12 – Education, communication, training, and public awareness**	Mass media campaigns on dangers of smoking and benefits of quitting	Bauer et al. [162]<br>Nierderdeppe et al. [163]<br>Weiss et al. [164]<br><br>Brinn et al. [165]<br><br>Flynn et al. [166]<br>Bauer et al. [162]<br>Farrelly et al. [167]<br>Niederdeppe et al. [163]<br>Wakefield & Chaloupka [168]<br>Goldman & Glantz [169]<br>Siegel & Biener [170]<br>Emery et al [171]	Prevention of smoking initiation among youth<br><br>Mass media campaigns most effective at preventing smoking among youth when conducted over a minimum 3 year period<br><br>Decrease in youth smoking prevalence	Strong but limited to research in high-income countries

FCTC Article	Policy evaluation	Source	Key findings on policy impact	Overall evidence for policy effectiveness
		Sly et al. [172]<br>Farrelly et al. [173]<br><br>Popham et al. [174]<br>White et al. [175]	Promotes anti-tobacco attitudes/beliefs among youth<br><br>Promotes smoking cessation among youth	
**Article 13 – Tobacco advertising, promotion, and sponsorship**	Comprehensive TAPS ban	ITC Project [130,131]<br>Caixeta et al. [132]<br>Harris et al. [133]<br>Kasza et al. [134]<br>Li et al. [135]<br>McNeill et al. [136]<br><br>Saffer & Chaloupka [137]<br>Blecher [138]<br>Saffer [139]	Reduction in public exposure to tobacco marketing<br><br>Decrease in tobacco consumption	Strong
	POS advertising and promotion ban	National Cancer Institute [140]<br>Paynter & Edwards [141]	Prevention of smoking initiation among youth	
	POS display ban	Spanopoulos et al. [142]<br>McNeill et al. [136]<br>Scheffels & Lavik [143]	Prevention of smoking initiation among youth	
**Article 14 – Demand reduction measures concerning tobacco dependence and cessation**	Advice from physicians	West et al. [144]	Promotes smoking cessation	Strong
	Training on cessation techniques for health professionals	Carson et al. [145]<br>Warren et al. [146]	Increase in provision of cessation treatments among trained professionals; increase in quitting among advised patients	
	Use of NRT and stop smoking medications	West et al. [144]<br>Cooper et al. [147]<br>Kasza et al. [148]	Increase in quitting among smokers who use NRT, buproprion, varenicline, cytisine, or nortriptyline	
	Telephone-based quitlines	Pine-Abata et al. [79]<br>Ossip-Klein & McIntosh [149]<br>Stead et al. [150]<br>Anderson & Zhu [151]<br>West et al [144]	Increase in quitline usage and quitting behaviour	

FCTC Article	Policy evaluation	Source	Key findings on policy impact	Overall evidence for policy effectiveness
		Miller et al. [152]<br>Abdullah et al. [153]		
	Behavioural cessation support	West et al. [144]<br>Bauld et al. [154]<br>Dobbie et al. [155]	Increase in quitting	
	Integration of cessation interventions	Mullen et al. [156]<br><br>Gibson et al. [157]<br><br>Ferguson et al. [158]<br>Judge et al. [159]<br>Brose et al. [160]	Increase in long-term quitting, and reduction in mortality risk<br><br>Increase in smokers' quit attempts, use of treatments, and short-term abstinence<br><br>Increase in quitting	
	Cost coverage for stop smoking medications	Cooper et al. [147]<br>West et al. [161]	Increase in use of stop smoking medications among smokers who tried to quit	
**Article 15 – Illicit trade in tobacco products**	Comprehensive measures to interrupt supply chain from manufacturers to illegal markets	HM Revenue & Customs [186]<br><br>Joossens & Raw [187]<br>Joosens [188]	Decrease in illicit cigarette trade<br><br>Reduction in market share of contraband cigarettes	Limited
	Track and trace systems to monitor production and distribution chain	World Health Organization [33]	Decrease in illegal production of cigarettes; increase in seizures of illegal tobacco products	
**Article 16 – Sales to and by minors**	Ban sale of tobacco to minors	Huang et al. [176]<br>Levinson & Mickiewicz [177]<br>Tutt et al. [178]<br>Verdonk-Kleinjan et al. [179]<br><br>Jason et al. [180]<br>DiFranza et al. [181]	Decrease in youth access to cigarettes<br><br>Reduction in youth smoking	Strong but limited to research in high-income countries
	Raising legal age of purchase	Staff et al. [182]	Reduction in youth smoking	

**FCTC Article**	**Policy evaluation**	**Source**	**Key findings on policy impact**	**Overall evidence for policy effectiveness**
	Implementation of measures to limit youth access to tobacco products in conjunction with other tobacco control programmes/policies	Gendall et al. [183]<br>Friend et al. [184]<br>Lantz et al. [185]	Prevention and reduction of tobacco use among youth	
**Article 17 – Provision of support for economically viable alternatives**	Alternative crop programmes	Akhter et al. [189]	Lower food security for tobacco farmers vs. alternative crop farmers	Limited
		Li et al. [190]<br>Kibwage et al. [191]<br>Kenya Tobacco Control Research Group [192]<br>Magati et al. [193]<br>Keyser [194]<br>Keyser & Lungu [195]<br>Molla [196]<br>Vargas & Campos [197]	Increase in net profit for alternative crop farmers vs. tobacco farmers	
**Article 18 – Protection of environment and health of persons**	Use of personal protective equipment	Ballard et al. [198]<br>Hipke [199]<br>Gehlbach et al [200]<br>Ghosh et al. [201] [202]<br>Arcury et al. [203]<br>Onuki et al. [204]	Prevention of green tobacco sickness among tobacco workers	Limited
	Education campaigns on risks of tobacco harvesting	Ballard et al. [198]	Increase in incidence of hospital-treated green tobacco sickness	
**Article 19 - Liability**	Use of FCTC in legislation and litigation	Muggli et al. [52]	Reference to and/or incorporation of FCTC into legislative objectives, definitions, and/or substantive provisions by at least 51 jurisdictions; reference to FCTC in litigation by at least 27 jurisdictions	Strong

FCTC Article	Policy evaluation	Source	Key findings on policy impact	Overall evidence for policy effectiveness
		McCabe Centre for Law and Cancer & CTFK [205]	Use of FCTC to defend against legal challenges to tobacco control measures; require governments to implement stronger tobacco control measures; and defend tobacco control measures in international trade and investment challenges	
**Article 20 – Research, surveillance, and exchange of information**	Regular monitoring of tobacco use	Dubray et al. [10]	Decrease in tobacco smoking over time	Limited
**Article 21 – Reporting and information exchange**	n/a	n/a	n/a	None to date
**Article 22 – Cooperation in the scientific, technical, and legal fields and provision of related expertise**	n/a	n/a	n/a	None to date
